# Redesign methodology for mechanical assembly

**DOI:** 10.1007/s00163-017-0255-6

**Published:** 2017-04-03

**Authors:** AbdulRahman El-Nounu, Atanas Popov, Svetan Ratchev

**Affiliations:** The University of Nottingham, Nottingham, NG7 2RD UK

**Keywords:** Design for assembly, Redesign for assembly, Aerospace assembly

## Abstract

Design for assembly is the concept of carrying out critical thought early in the design stage to create assembly easement at the production stage. In the aerospace industry, products have very long lives, frequently being optimised rather than introducing new products. This has meant older products, which are stable income generators, have not benefited from the latest design for assembly methods and manufacturing technology suffers from obsolescence. It has been established that a large percentage of overall product cost is determined at the design stage; thus, existing products suffer from preloaded costs. This paper takes existing design for assembly methodologies and analyses them with respect to the unique challenges involved in legacy product redesign. Several novel factors that contribute to redesign analysis are identified such obsolescence impact and a holistic operation difficulty assessment. A tool is developed to identify potential redesign for assembly projects. The tool is demonstrated through the application of real data and comparing against business decisions. The tool was found to provide a strong indication of where profitable projects may be launched.

## Introduction

The aircraft industry produces long life products that stay in production for decades. The two dominant commercial aircraft manufacturers, Airbus and Boeing, rely on their single aisle, shorter range products as consistent revenue streams. The Airbus A320 first flew in 1987 and the Boeing 737 first flew in 1967 (Norris 2014). To reduce risk, these companies have adopted an iterative development approach to their products as opposed to regular new product introduction. This approach has been vindicated by the record sales achieved by the latest iteration of the Airbus A320, known as NEO (Airbus.com 2014). With an extensive order book secured, the company has now shifted its focus onto cost reduction to maximise profits and to accommodate rises in other costs, such as inflation. One area that has been targeted is manufacturing and assembly. Manufacturing systems in long life products are at risk of being superseded by competitors if they are not addressed regularly. Technology insertion is typically the first approach to manufacturing system optimisation (Arc Advisory Group 2011), but there comes a point when the product also requires optimisation for manufacturability. Identifying where design for manufacture and assembly optimisation projects can occur on a product that has been in production for decades is a difficult task. Most design for assembly methods is optimised for new product introduction of small volume assemblies. This paper presents a redesign for assembly toolset that includes novel factors, such as obsolescence impact and operation difficulty. The aim of the tool is to give the engineer a process comparison mechanism that enables the identification of projects related to redesign for assembly that are potentially profitable.

## Literature review

Design for assembly and manufacture (DFMA) is a method that has been widely adopted in the development of new products. The terms “Design for Manufacture” mean the design for ease of manufacture of the collection of parts that will form the product after assembly. The terms “Design for Assembly” mean the design of the product for ease of assembly (Boothroyd et al. 2014). Before the adoption of DFMA, design engineers would typically work independently of manufacturing and assembly engineers. Boothroyd et al. (2014) state that over 70% of product cost is assigned at the development stage. Indeed, the objective of DFMA is to reduce cost in the overall life of a product. There are a number of approaches in the literature on how this can be achieved. Most notably, Boothroyd et al. (2014) were a pioneer in formalising such an approach. They provide three criteria upon which each part must be examined. They are as follows:

1. During the operation of the product, does the part move relative to all other parts?
2. Must the part be of a different material than or isolated from all other parts already assembled?
3. Must the part be separated from all other parts already assembled because otherwise necessary assembly or disassembly of other separate parts would be impossible?

The application of this approach is demonstrated extensively by Boothroyd et al. (2014). However, the applications are all of small volume parts with tolerances that are more relaxed than those required in the aerospace industry. In addition, the Boothroyd method is focused on part count reduction. Frey et al. (2007) carry out an in depth analysis on the effect of part count reduction in increasing product robustness. As part of this analysis, they state that although part count reduction can lead to a reduction in cost; in high value components, it can also lead to an increase in cost. The examples presented by Boothroyd are of relatively low value assemblies and reducing part count usually involves transferring part functionality into other components making their manufacture slightly more complex. If this approach was implemented on aircraft components, then this would likely result in unmanageable escalating costs. In a redesign scenario, reducing part count is even more difficult. In the case of legacy aircraft, the infrastructure involved in manufacturing the components would also need to be updated. Suppliers of complex machined components would be required to increase their capability to accommodate additional functionality of components. Fixtures and assembly technology would also need upgrading. In this light, part count reduction is only part of a strategy to reduce assembly costs in redesign of aircraft assemblies.

Another approach to Design for Manufacture and Assembly is that of Miyakawa and Shigemara (1986). They developed the Hitachi Assemblability Evaluation Method (AEM); a tool that feeds into design to improve producibility of the product. It consists of two steps: the first is to assign quantitative data to existing assembly processes and the second is to sum these values to create an AEM score. This is then used to assess time and cost. Ohashi et al. (2002) further expanded on this method by introducing part-based cost estimation. Similar to this is the Lucas method, developed by Sealy and Corns (1992). They assigned ratings to the following:

- Functional analysis: interrogation of every component as to its function within the product;
- Handling analysis: examination of the ability to handle and position each part to achieve the correct assembly orientation;
- Fitting analysis: assessment of ease of holding an assembly.

In a redesign context, it is difficult to make accurate estimates of cost impact due to the associated impact on delivery disruption, supplier capability development, and in house infrastructure development. In addition, at redesign stage, the selection of processes and technology has already taken place and decisions on technology and process selection will also include the cost of replacing infrastructure against the cost of keeping functional existing infrastructure.

Hsu and Lin (2002) also produced a quantitative method. This method was designed to assess component accessibility during assembly. They argue that accessibility is an indicator of the probability of successfully achieving design targets within manufacturing capability. This method relies on the availability of 3D CAD models to carry out assessments. In the case of legacy aircraft, the assemblies being considered for redesign are several decades old, developed before the widespread application of 3D modelling. Furthermore, the accessibility being discussed is access to the part, or difficulty in placing components into the part. Accessibility in aircraft assembly is different; the challenges are those of line of sight and ergonomics due to the size of the assembly.

Whitney (1988), in the Harvard Business Review, comments on DFA in the context of manufacturing by design. He says that value engineering and design for producibility are the real objectives when embarking on a DFA journey. He says that DFA cannot achieve fundamental improvements, because it considers the product as a collection of parts instead of something to satisfy larger goals such as reducing costs over the life time of the product. This further emphasise the point made about part count reduction in aircraft assembly.

There is little specific literature carried out on redesign for assembly. Fabricius (1994) asserts that the traditional method for assembly improvement is through technology insertion. This eventually reaches a saturation point due to inflexibility and high capital costs. He proposes a seven step procedure for design for manufacture. Although he asserts his method is suitable for redesign, his procedure is more in line with initial development of products then redesign. It starts with the production of conceptual ideas that are refined at every step. This cannot be implemented in a redesign scenario.

Desa (1987) acknowledges the importance of redesign. He developed a tool set and interface module that relies on historical knowledge of the original design for input into redesign. He states that the following aspects are critical when carrying out redesign:

- good performance measures;
- good understanding of the effect of design modifications on these performance measures;
- procedures that utilise the first two points.

Desa’s focus, much like Boothroyd, is on small assemblies evidenced by his frequent reference of a small electromechanical assembly. Furthermore, Desa presents no real case studies, merely a theoretical demonstration. In addition, he does not refer to the nuances in deciding which redesign projects to go forward with. An aircraft assembly will have many areas that may benefit from redesign, but not all can be achieved; the selection of which project to target is a difficult task.

Lee et al. (1993) proposes using the traditional DFMA with some modifications for redesign situations. The two modifications he proposes are the inclusion of analysis of assembly sequence as part of redesign DFA methodology and also the consideration of design rationale in the same process. He developed what he called the Backward Assembly Planner (BAP) to identify assembly problems and REVerse ENGineering (REVENGE) toolset to come up with assembly solutions. He argues that his methodology leads to a more realistic DFA outcome as the activity of assembly planning is coupled tightly with redesign. Hsu et al. (1993) also proposed a method that uses assembly planning as an input. In long life products, extensive consistent assembly planning and optimisation already takes place. It is an easy target for optimisation as heavy investment is not usually required.

Lefever and Wood (1996) states that part count reduction is the most effective Redesign DFA target. As mentioned previously, although this may be affective in some cases, directing redesign efforts towards one aspect is not an efficient method of addressing assembly issues. A more comprehensive analysis of the product and manufacturing system in place is more efficient.

All of those methods fail to address a critical aspect of aircraft production. This is that the products themselves are of long life. There is no account of obsolescence of technology and processes in the analysis. In addition, there is no method to holistically assess assembly process difficulty. Usually, one aspect of process difficulty is addressed, such as accessibility or handling.

In a report written by Peter Reynolds for the Arc Advisory Group (2011), who are a global market research advisory group, it is stated that there is an estimated $65 billion of obsolete automation technology within the manufacturing industry. Obsolescence in manufacturing, especially in aircraft manufacturing, takes a different definition than is typical. Sandborn (2007) defines obsolescence as “the loss or impending loss of original manufacturers of items or suppliers of items or raw materials”. Suppliers to aircraft manufacturers are unlikely to discontinue a product or service to aircraft manufacturers as this will cutoff a potentially long lasting revenue stream. However, this does not stop the technology industry itself from developing superseding technologies or processes. Obsolescence in this case is that a competitor gains an advantage by deploying the latest technology and processes. Indeed, this kind of obsolescence can exist in the same company, where a more recent product employs more efficient transferable processes.

## Redesign scenario

This particular research is focused on the assembly of a wing box structure. The wing is part of the shorter range aircraft family and is approximately 15 m in length. As mentioned previously, this family of aircraft has been in production for several decades. Company policy throughout the life of the aircraft has been to introduce design changes to the product to improve product performance. This policy has been justified with the volume of sales generated in recent years.

As for optimising manufacturing and assembly, small-to-medium-scale technology insertion has been the typical solution for enhancing the production system. Changes in design to create manufacturing easement have been difficult to achieve due to the challenge in demonstrating an attractive business case. The high cost of carrying out design changes and the potential for recertification costs is prohibitive. In addition, the reward for decreasing manufacturing costs is typically not comparable to the reward from sales and performance savings achieved through performance modifications. The success of the single aisle aircraft market is also a factor as competitors adopt a risk averse attitude so as not to compromise delivery of aircraft and thus lose market share.

As mentioned before, in more recent years, sales of these aircraft have been numerous and decreasing delivery lead time has become a more prominent issue. Therefore, manufacturing and assembly system improvements are being demanded by the business and the introduction of design changes for assembly easement is viewed more favourably. Continuous product development teams are now encouraged to pursue them.

As discussed, DFA methodology is not entirely suited for this particular context. DFA methodology has traditionally focused on new products that are small in volume. The challenges in this scenario are summarised as follows:

- large volume components;
- high tolerance assembly criteria (positional accuracies of holes at 0.2 mm);
- long life products;
- high capital monument technology and tooling already in place;
- uncompromising delivery targets;
- knowledge disconnect.

### Large volume components

Components require specialised tooling for lifting and handling. Accessibility is also an issue due to the components limited line of site. Operators are sometimes working on assemblies without having visual sight to all areas being affected. There are also times when the operator has to work inside closed boxes and cramped spaces. These aspects do not form part of the critical thinking in small volume assemblies.

### High tolerance assembly criteria

Due to the large safety requirement for aircraft, assembly tolerances are high. Coupled with the large volume of aircraft, this makes the costs of associated assembly technology high. This aspect is one of the advantages that can be gained through careful redesign analysis.

### Long life products

This is the largest factor in creating assembly issues when competing against rivals. Although the processes that are in place are functional, in many instances, there are likely to be superior options available. For example, new aircraft programs, such as the Airbus A350 and the Boeing 787, are likely to have developed new assembly processes that may be backward compatible. Similarly, the technology used during assembly has likely been superseded throughout the years.

### High capital monument technologies

Expensive bespoke technology has already been invested in, such as high accuracy fixtures and bespoke monument automation. In addition, problematic is that datum structures for legacy products typically reference high accuracy fixtures.

### Uncompromising delivery targets

There is a cultural reluctance to introduce any changes that may put the production system at risk of not meeting its immediate delivery targets. With performance improvements, this risk is offset by the estimated revenue generation. In the realm of production system improvement, as mentioned before, redesign for assembly, up until very recently, had been considered a major risk. Even technology insertion, which has been the predominant method of improving the production system, is carried out in a manner, where only small-scale step changes are introduced. Further to this, the introduction of new technology or processes would entail training and ramp up costs and delays that would be viewed unfavourably.

### Knowledge disconnect

Another interesting element contributing to a lack of redesign for assembly activity is knowledge on where to carry out projects. As mentioned, the business is interested in exploiting any opportunity to increase production rates and reduce costs. When continuous improvement teams attempt to identify areas or projects that would benefit from a redesign analysis, they rely on information from production teams. This researcher carried an investigation with members from different production teams. They were asked what changes to the product could yield an assembly improvement. Most suggestions were minor changes that would yield small savings that although not insignificant and would not justify the cost of design changes.

Thus, it becomes apparent that a method for identifying where redesign activities is needed on legacy products and production systems would be of benefit.

## Redesign methodology

The method is required to carry out aspects of traditional DFMA methods but also account for its application on existing production systems. The method is designed to streamline the project selection process and is listed as follows:

1. data acquisition;
2. analysis;
3. idea generation;
4. selection and approval;
5. implementation.

The methodology follows a linear flow. Structured data gathering are the first step, followed by knowledge generation that leads to solution generation and implementation. The first two stages of the flow are the creation of knowledge. After knowledge is generated, solution generation becomes the target. The aim of this method is not to generate solutions, rather the aim is to highlight where redesign for assembly projects may be profitable. Corbett and Crookall (1986) highlight that rigid solution engineering techniques are not the most effective way of producing engineering results. Rather, engineers should be allowed to apply their own ingenuity and creativity in finding solutions. This is the approach adopted by this methodology.

Whilst engineers should perhaps typically be left to create solutions through uncontrolled innovation, the selection and approval process inherently plays a critical part in the project progression. Frustratingly, it is not always the optimal solutions that are selected for implementation due to circumstantial prohibitors, such as simultaneous projects, company direction, or even negative company politics.

**Fig. 1:**
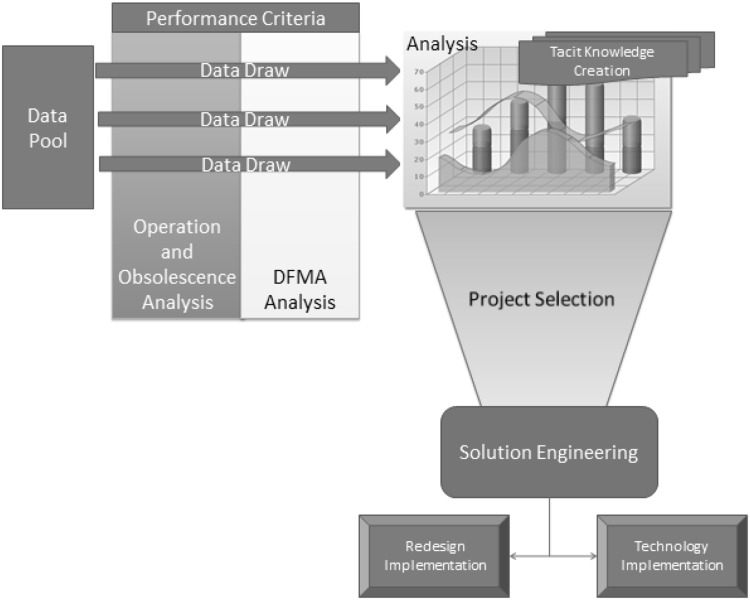
Redesign for assembly method

The methodology is summarised in Fig. 1.

The first phase of this Methodology is the identification and collection of data and the creation of a data pool. These critical performance criteria are made up of two categories. The first category is measureable criteria related to traditional DFA. The second category is factors that are unique to products that already in production.

Once a pool of data is created, the next step is data mining. In any given investigation, certain criteria will take precedence depending on the objective of the study. For example, if reducing assembly time is a current objective, then indicators that provide information on time and efficiency will take precedence. At this stage, the most relevant performance criteria are selected to act as a data filter. Once this takes place, effective data can be drawn from the previously assembled data pool. This methodology is unique in that it considers manufacturing enablement through design after a product is in steady-state production. The methodology takes into account both traditional design for assembly techniques as well as specific factors related to redesign for assembly. As mentioned previously, traditional DFMA techniques tend towards initial development of products through concurrent consideration of design, manufacture, and assembly. Redesign for assembly aims to do this whilst also acknowledging the restrictions that a steady-state product has to navigate through and also the advantages in available data that a live product provides.

Analysis of this filtered data leads to the creation of recorded tacit knowledge. This is defined by Groff and Jones (2003) as personal knowledge embedded in individual experience. It involves intangible factors, such as opinions, perspective, and values. This type of knowledge is more difficult to record and transfer.

The creation of knowledge in turn leads to the project selection stage. Depending on a company’s current strategy, projects will be selected according to the most valuable criteria at that time. After project selection, the projects are taken into development stage, named the Solution Engineering phase. As discussed, solution engineering is not the goal of this method, but it needs to be acknowledged. It is intended that the analysis phases, specifically the obsolescence analysis, will provide a strong indication of a potential project direction.

## Tool creation

From the previously discussed methodology, the most critical aspect is the section, where data are drawn through the performance criteria. Therefore, the development of a tool to implement the stated methodology in that area is proposed. The tool will provide quantitative results to compare processes against each other. Therefore, the first task in putting together such a tool is to identify measurable factors that can provide data.

Four groups were approached separately in pursuit of this criteria as well as also asking them about their thoughts on potential DFA projects in the hope that some consistent parallels could be drawn from each group. The four groups were as follows:

- Operations and manufacturing engineers: these are directly involved in manufacturing improvement projects and day-to-day running of production;
- Industrial architects: this is a body that attempts to link manufacturing improvement with engineering improvement;
- Engineering continuous improvement: these are engineers that typically work on performance improvement for the aircraft;
- Research and technology: this is a team that develops assembly and manufacturing technology and processes.

These are all separate roles and members of each function do not assume responsibilities of other functions.

Four main aspects emerge from Table 1. These are as follows:

**Table 1:** Showing the stakeholders engaged to define performance criteria

Group	Feedback
Operations and manufacturing engineers	Cost and failure are the main drivers to target. Suggested projects were small-scale projects that would not payback after a design change. An example was certain features being too close to a work area prohibiting the use of a faster tool
Industrial architect	Cost, failure and also performance improvement were projects that the Industrial architect is interested in
Engineering continuous improvement	Aircraft performance improvement were key for any project in this team, some isolation from manufacturing was prevalent with manufacturing projects only considered as part of larger engineering improvement projects
Research and technology	Technology obsolescence and recurring cost improvement were the main drivers behind projects in the Research team

- failure analysis;
- cost;
- operation difficulty;
- market changes and obsolescence.

### Failure analysis

Failure is anything during manufacture phase that contributes to further unplanned costs. Through this investigation, it was observed that failures that affect the performance of the product are most critical, and thus, they are taken most seriously. As such these failures go through analysis to eliminate recurrence or solutions are engineered in advance to address them before they occur. However, as per the definition stated previously, these are not all failures that are interesting to this investigation. This tool will attempt to capture those failures through engaging with the specific operators and manufacturing engineers that work in that process. The most direct way of capturing this data is through disruption time. However, this is not easily accessible as disruption time is classified into several categories and those categories are presented as time figures that capture other areas of disruption. Therefore, failure will be captured in the operation difficulty assessment as a question that is answered through feedback from the operators. This is shown in Table 4.

### Cost

In any manufacturing business, operations are mapped out in great detail with time allocations identified. There are three main inputs that can provide useful data for this analysis. They are as follows:

- Work content tracker: captures operation order and flow as well as the number of operators assigned to work on particular tasks;
- Standard time intervals, man hours, and number of operators: captures all time-related aspects of an operation;
- Critical path: captures the operations that lie on the critical path of production.

Cost carries the heaviest weighted factor that will feed into this model. Depending on the scenario that the program is operating in different weight allocations will be given to hours that can potentially be saved. If, for example, the company has an overall drive to reduce the total number of man hours during wing assembly, then any hour that can be saved becomes critical. If, however, the company is looking to increase production rates, then special emphasise will be placed on hours that lie on the critical path. Three measurable will be used to compare the impact of cost on different processes. They are:

- overall number of hours allocated to the completion of a process;
- critical path time;
- number of operators involved in a single assembly process.

### Operation difficulty

The following methodologies have been developed in the past with a view to operation difficulty:

- handling score (Hitachi Assemblability Method);
- number of parts and nature of activity (Boothroyd and Dewhurst);
- assembly plan evaluation (Hsu);
- accessibility (Hsu and Lin).

A method to determine a quantitative number that can provide an indicator to operation difficulty using an adaptation of these methodologies is required. The proposed method is developed specifically for wing box assembly in a large commercial aircraft manufacturer. In order for it to be used in any alternative capacity, the questions that are asked will need to be tailored for that assembly environment. The main assembly activities are categorised and analysed in terms of complexity. The main operations that wing box assembly consists of can be categorised as follows:

- clamping;
- drilling;
- sealing;
- bolting;
- cleaning;
- measuring/checking.

The way the analysis has been set up is that each operation is raised and the complexity of the operation is assessed based on a series of questions. Each set of questions provide a number that are summed together. A higher number indicate a higher operational complexity or difficulty. As well as these operation specific questions a number of overall operation questions. These questions take into account the total number of components involved in the activity and the total number of operations. This tool also assesses the level of value being added by the operation. For example, a measurement operation or a cleaning operation is essential but could be better served with an embedded solution or a more efficient process in the case of cleaning. The more variety involved in a process, the higher the operation difficulty number will be. The measurables that are collected from this aspect are as follows:

- Operation Difficulty Score;
- number of components;
- number of process actions.

### Market changes and obsolescence

This appears to be the main contributing factor for a steady-state production system. The amount of saving or cost opportunities that are not exploited due to obsolescence is immeasurable.

Obsolescence in this context is process obsolescence. As mentioned previously, suppliers are unlikely to stop providing a product or service required by a large OEM, this is especially true in a long life product, such as an aircraft. This does not mean that they will cease to develop better products. The moment a competitor takes advantage of a manufacturing technology that is superior to the one currently employed that technology begins to become obsolete. The extent of that obsolescence depends on the level of advantage provided. This at first seems like technology insertion with no place in a DFA analysis. However, these researchers have encountered multiple instances, where an available technology that can provide significant improvement to assembly is discarded, because the current product design is not able to accommodate that technology. This is one aspect of obsolescence.

The second aspect of obsolescence is a knowledge management recirculation issue. It would be prudent for manufacturers to superimpose their new processes on their older products.

In this analysis, it is difficult to record these data quantitatively. The researcher proposes a gated classification process to categorise the level of obsolescence of the process under analysis. The following defined Obsolescence Impact (OBI) Factors are proposed:

It should be noted that the improvement, mentioned in Table 2, is defined as whether the process or technology has been superseded elsewhere to mature level.

**Table 2:** Obsolescence Impact Score criteria

OBI 1
Process or technology is State of the Art and is being utilised efficiently
There are no updated techniques for this process and no visible opportunities for improvement
OBI 2
The technology or process is still current and serves its purpose well however there are potential improvements being developed
There are immature technologies being developed but their capability is unknown
OBI 3
Immature technologies or processes have been developed outside of the business but it is unknown how effective they will be
The process or technology is still functional and is not considered a risk in the immediate future
OBI 4
Technology is out dated and whilst it functions correctly there are mature off the shelf solutions that can improve the process
Processes have been superseded outside of the business but have not been demonstrated specifically in the targeted business area
Processes have not been superseded inside of the business
If this process is not addressed it will become a bottleneck in the future
OBI 5
The process or technology is out of date
The process is a bottle neck in the manufacturing assembly line
Mature processes have been developed and implemented to address this area in other parts of the business
Mature technologies are currently being used by competitors for this process or a process very similar to it

It is important to note that the quality of the benchmarking exercise is critical in determining an accurate OBI score.

### Redesign for assembly tool

With these criteria in mind the tool is demonstrated in the following. The proposed tool was applied to the scenario of the installation of brackets that are bolted onto the structural stiffeners of an aircraft wing skin and to the wing ribs. Due to the confined nature of where they are installed, the use of the latest drilling machines is unavailable. The values shown are not real values for confidentiality reasons. However, the conclusions drawn are based on the real values and are still valid.

## Case study

### Setting the problem

**Fig. 2:**
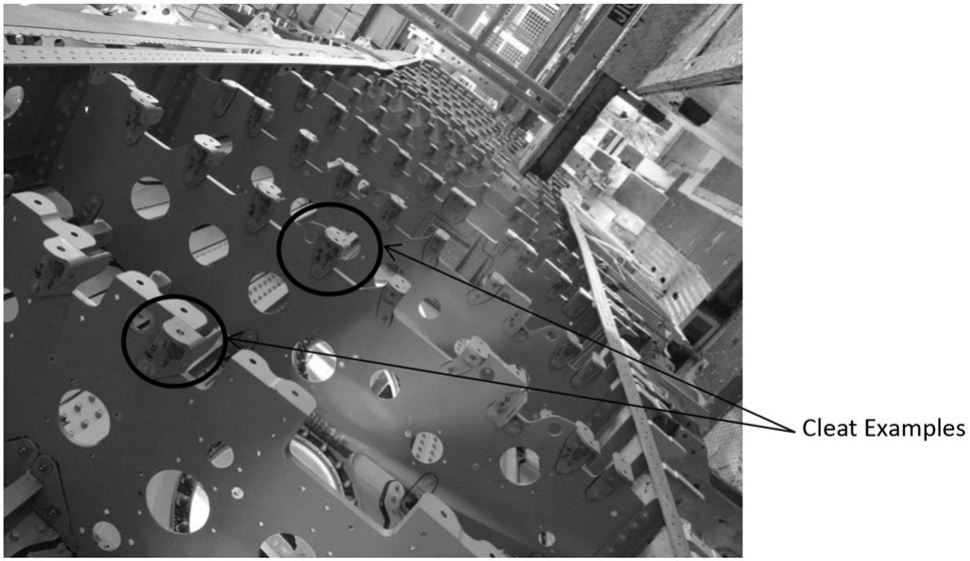
Showing Butterfly brackets bolted onto wing ribs

**Fig. 3:**
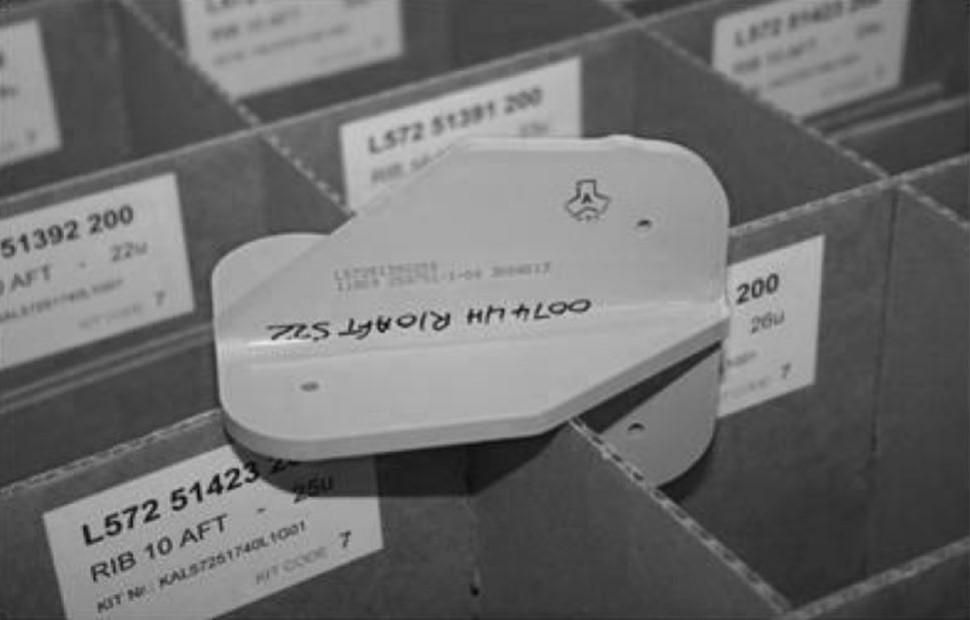
Showing a cleat bracket

To test the methodology being proposed in this work, a case study with a known outcome is used. The case study looks at the installation of a large number of butterfly brackets, known as cleats. The problem has been persistent despite several attempts to find solutions through technology. Recently, a proposal to design out these butterfly brackets has been accepted and when completed will offer considerable assembly savings. As this case has a known redesign for assembly outcome, it provides an ideal case study to measure the effectiveness of the tool. The location of these cleats is shown in Fig. 2, and an example of a cleat is shown in Fig. 3.

The wing box is assembled in a section during production labelled Stage01. This is consistent for the Single Aisle, Long Range and A380 aircraft. In general, the majority of required holes are now drilled using a semi-automated drilling process using a drilling unit called an automated drilling unit (ADU). This process involves the use of thick templates that act as fixtures for the drilling unit to drill a hole with a countersink in one single operation. Previously, to drill a hole to the required tolerances, a multi-step progressive drilling method was used that involved the following steps:

- pilot hole;
- first size;
- down size;
- reaming;
- countersink cage.

With the introduction of automated drilling units (ADU), cycle time has been reduced dramatically by removing several drilling passes. These ADU are used to carry out the majority of drilling operations on areas that have large collections of holes.

There are some areas that did not benefit from the deployment of ADUs. One such area is cleat installation. Cleats are butterfly brackets that are bolted onto the structural stringers on the wing skin and to the wing ribs. These brackets offer extra support, strengthening the interface between the Wing Skin and the Ribs. Due to the confined nature of where they are installed, the use of current ADU is unavailable. Therefore, the process of drilling these butterfly brackets has remained as before.

#### Cleat drilling process

The cleat installation process is listed as follows:

1. collect equipment;
2. ensure work area is clean and clear;
3. check and mark edge distances as per drawing;
4. clamp cleat to stringer;
5. pilot forward rib hole and pin;
6. pilot stringer hole and pin;
7. pilot remaining holes and pin stringer hole;
8. open aft rib hole to requirement and check;
9. open stringer hole to requirement and check;
10. repeat for remaining holes;
11. remove cleat (unpin rib last);
12. de-burr;
13. remove swarf.

**Fig. 4:**
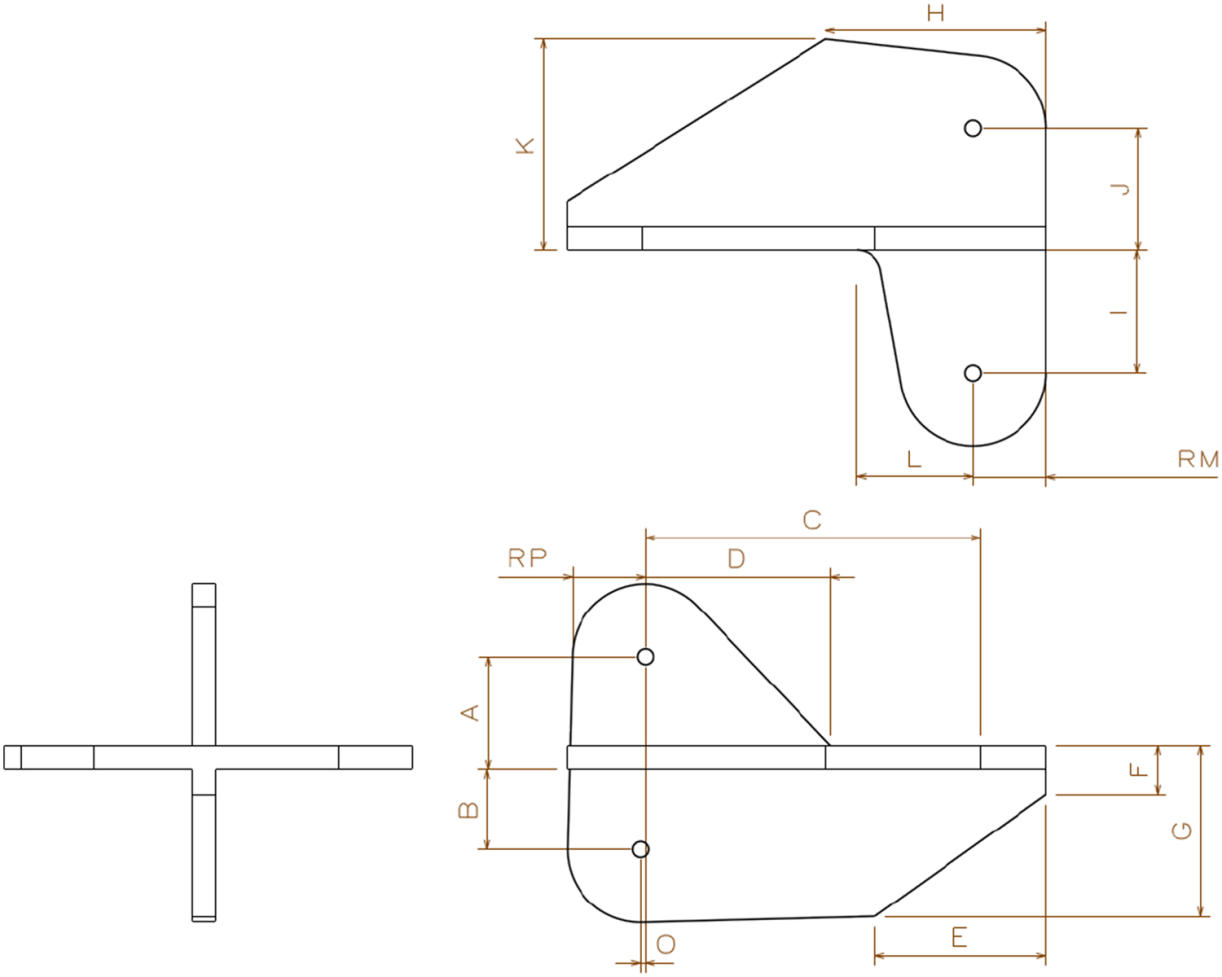
Showing a typical butterfly cleat

The process is complex, and the working conditions are confined. Pilot holes are drilled on the cleats during their manufacture. These holes are then transferred to the adjacent structure after locating and clamping individual cleats in position. As well as the many steps involved in drilling each hole of each cleat, there are a variety of cleats differing by shape, dimensions, and hole locations. Therefore, as well as the many drilling steps for each hole in each cleat, the hole positions vary on the cleats themselves. This adds an extra step on each drilling operation for measurement. In one particular aircraft, there are 123 cleat variations. In total, there are 150 cleats which are installed. Many of these cleats are similar in shape but have slight differences in dimensions. Many times dimensions vary from one cleat to the next by 0.1 or 0.2 mm. The most commonly occurring cleat shape is the butterfly shape, as shown in Fig. 4. In this shape alone, there are 42 different variations.

The number of variations in cleats is an example of the pre-DFMA culture during product development. Design engineers were following strict design rules driven by weight reduction targets. From the design engineers’ point of view, standardising the cleat shape would serve no purpose as at that time, every cleat would still need to be measured and installed. For the manufacturing engineer, the variation in cleat shapes and dimensions creates two problems. The first is that simplifying this task becomes very difficult if a solution is sought through technology insertion. Typically, implementing an automated or tooling solution requires a stable process with minimal variation lest the costs escalate and become unfeasible. The second is that this manual, variable task creates an environment, where errors are more likely to occur.

**Fig. 5:**
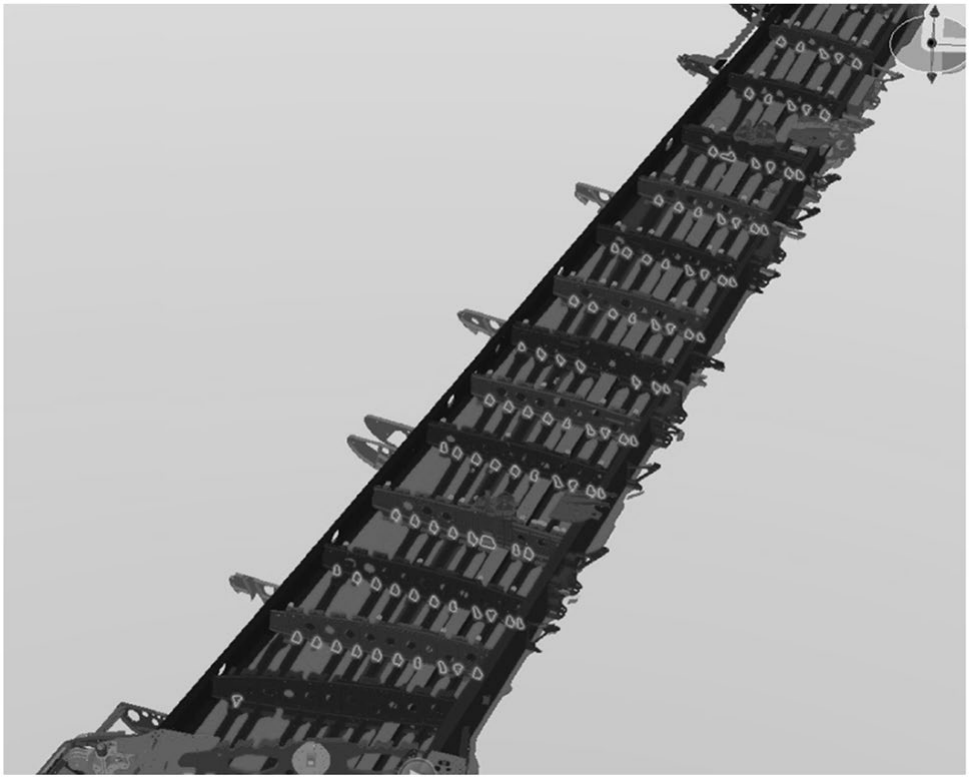
Visual demonstration of where cleats appear in a wing box

Due to the reasons mentioned, technology solutions would be very costly in this area, and thus, an alternative solution would be beneficial. The proposed tool is thus used to demonstrate that a DFA solution is a more interesting prospect (Fig. 5).

### Tool demonstration

#### Cycle time and cost

The cycle time and cost for this area are summarised as follows: five operators are required for a shift of 8 h to complete the full set (Table 3).

**Table 3:** Real values not shown

Drilling of brackets	Per wing set	Per single wing
Overall operation man hours	60	30
Critical path (h)	10	5
Number of operators	12	6

#### Failures

As mentioned previously, recorded data for failures is not captured in a method that can be used in this kind of analysis. The proposed method is to capture feedback from the operations teams for the individual work packages. The input into the proposed tool will be a positive or negative response. Failures and rework will appear as a single numeric in this tool; either they will be listed as an issue or they will not.

#### Operation Difficulty Score

The operation difficulty score is calculated using a set of questions displayed in Table 4. It should be noted that the series of questions are unique to this manufacturing system. A unique set of questions to achieve a operation difficulty score is required to be developed for every unique manufacturing system. For example, the questions shown in Table 4 are only applicable to the wing box assembly area. They are not applicable to the next stage of wing production; system installation.

**Table 4:** Operation Difficulty Score

Clamping operation	Is there a clamping operation	Is access required from both sides of work piece	Can the force being applied damage the work piece	Is more than one operator required to apply the clamp	Access: is the working area visible to the operator during the operation	Access: is the operator required to strain to reach the working area
	Yes	Yes	No	No	Yes	Yes

Drilling operation	Is there a drilling operation	Are drilling jigs required	Is more then one operator required to install the jigs	How many passes must be drilled before the final hole is produced	How often is a temporary fastener inserted	Access: is line of sight not available
	Yes	Yes	No	3	3	No

Sealant operation	Is there a sealant operation	Does cleaning need to take place	Access: is the working area visible to the operator during the operation	Access: is the operator required to strain to reach the working area		
	No	N/A	N/A	N/A		

Bolting operation	Is there a bolting operation	Is access required from both sides of the work piece	Can a single operator access both sides	Access: is the working area visible to the operator during the operation	Access: is the operator required to strain to reach the working area	
	No	N/A	N/A	N/A	N/A	
How many parts are to be assembled	150					
How many operations take place in total	3075					

Lifting	Can the part be lifted manually?	How many people are required to lift and position	Is a measurement using metrology equipment required?	Is a check required using a tool such go-no go gauge?		
	Yes	1	No	Yes		
Non value adding operations (such as cleaning) or parts not fitting	75	Fettling operations				
Operation Difficulty Score	13					

There are two notable numerical values that are gained in the analysis shown. They are the operation difficulty score and the number of operations. The operation difficulty score sums together the number of different types of operations, for example, if the operation involves a drilling, bolting, and sealing activity, these will sum together as 3. The operation difficulty score also takes into account the difficulty of those activities. For every further complication, an extra 1 is added to the operation difficulty score. For example, if a drilling operation is a single pass, this will merely count as one. However, if the drilling activity requires several passes, along with changes in tooling or cutter size, then this is a further complication and thus will be counted as 2.

The second significant numeric that appears in Table 4 is the number of actions that take place in the operation. This is a count of the number of actions that the operator is required to carry out to complete the package of work. For example, one drill cycle would count as one action. If, to complete a hole, several drill cycles are required, this would count as several actions. Together with the operation difficulty score, these two factors provide a top level and superficial view of the operation difficulty that can be used to make quick assessments.

### Obsolescence

As discussed previously, the obsolescence analysis is difficult to quantify. The author has proposed an obsolescence impact score that requires the user to make a judgement based on gathered data. The two streams of data that are required to be collected are “changes to the process or part that are present in more recent products” and a technological assessment of what is available throughout the industry. The first set of data is easier to acquire and is definitive. The second set of data requires an engineer with experience in the particular field of technology to carry out a small benchmarking analysis. The risk here is overlooking technology or not having exposure to a particular technology. The impact score is assigned from 1 to 5. An impact score of 1 means that the current process is not obsolete and an impact score of 5 indicates that there is room for possible improvement.

In this case, the score provided is 4 due to the acknowledgement that it falls under the OBI characterisation from Table 2 of “if this process is not addressed it will become a bottleneck in the future”. A benchmarking exercise on potential technology was carried out, and despite the passage of several decades, since this process was introduced, a cost effective technology is still not available.

The main reason the obsolescence impact score in Table 5 is high because of the development of new stress and structural analysis methods. These have enabled studies to take place that have allowed an aircraft in a different program to benefit from cleat standardisation. In addition, these methods have enabled an analysis that has indicated that cleat deletion is now also a possibility.

**Table 5:** Obsolescence Impact score

Internal factors		
Long range aircraft have standardised cleats		
New stress and structural analysis methods open the possibility for cleat deletion		

New technology developments	Maturity	Comments
Confined space robot: snake arm robot	Low	Prohibitive price and lack of effective payload capability
Advances in drilling capability	Low	

Obsolescence impact rating		
1–5		
1—little to no impact		
5—process or technology are highly outdated		
Assessment	4	

### Cleat analysis section

Table 6 shows the results that would be generated by the proposed tool and methodology. The problem with any acquired data is that there is nothing to compare it against, and therefore, they are currently meaningless. In order for the tool and methodology to be effective, more data are required to create comparison scenarios.

**Table 6:**
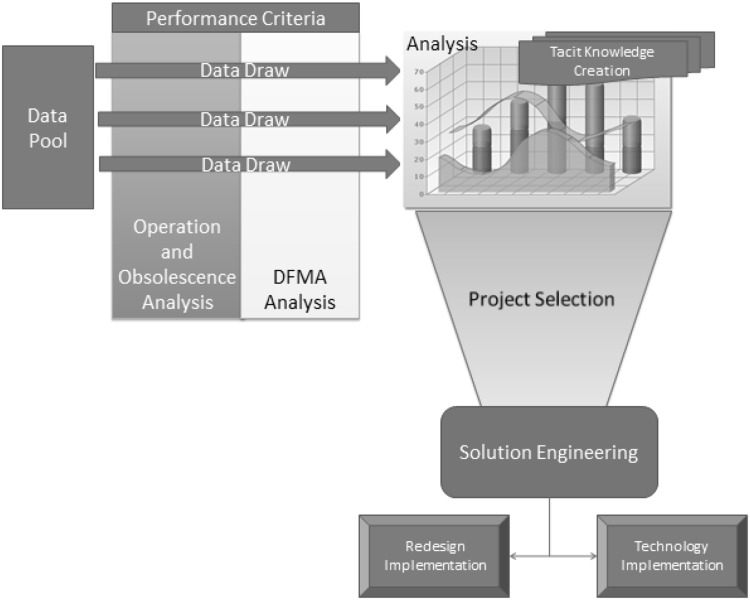

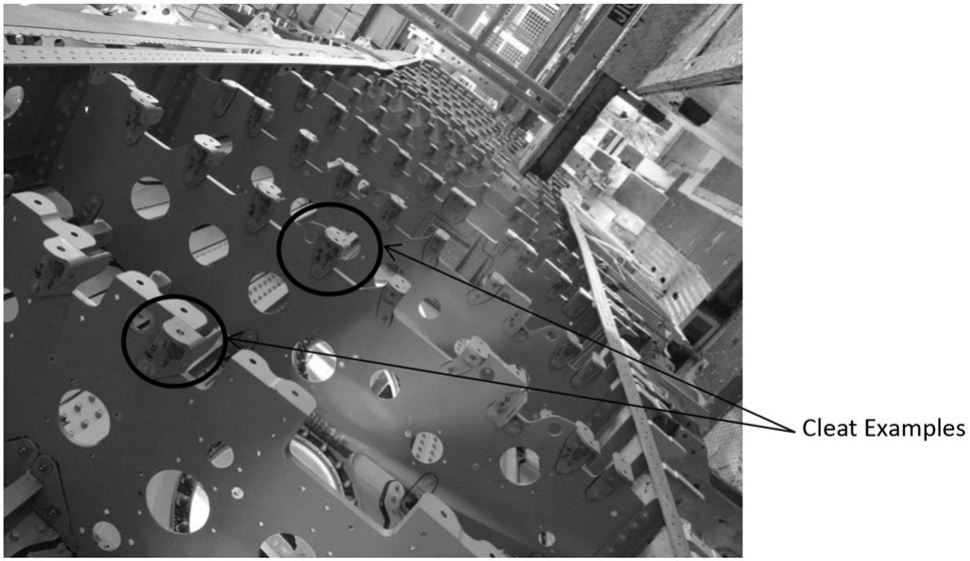

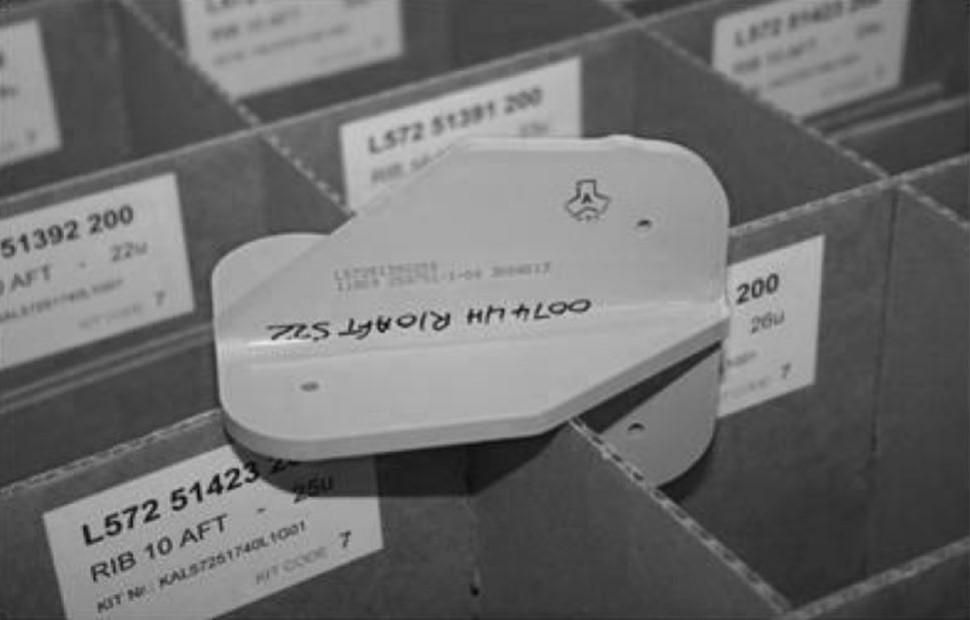

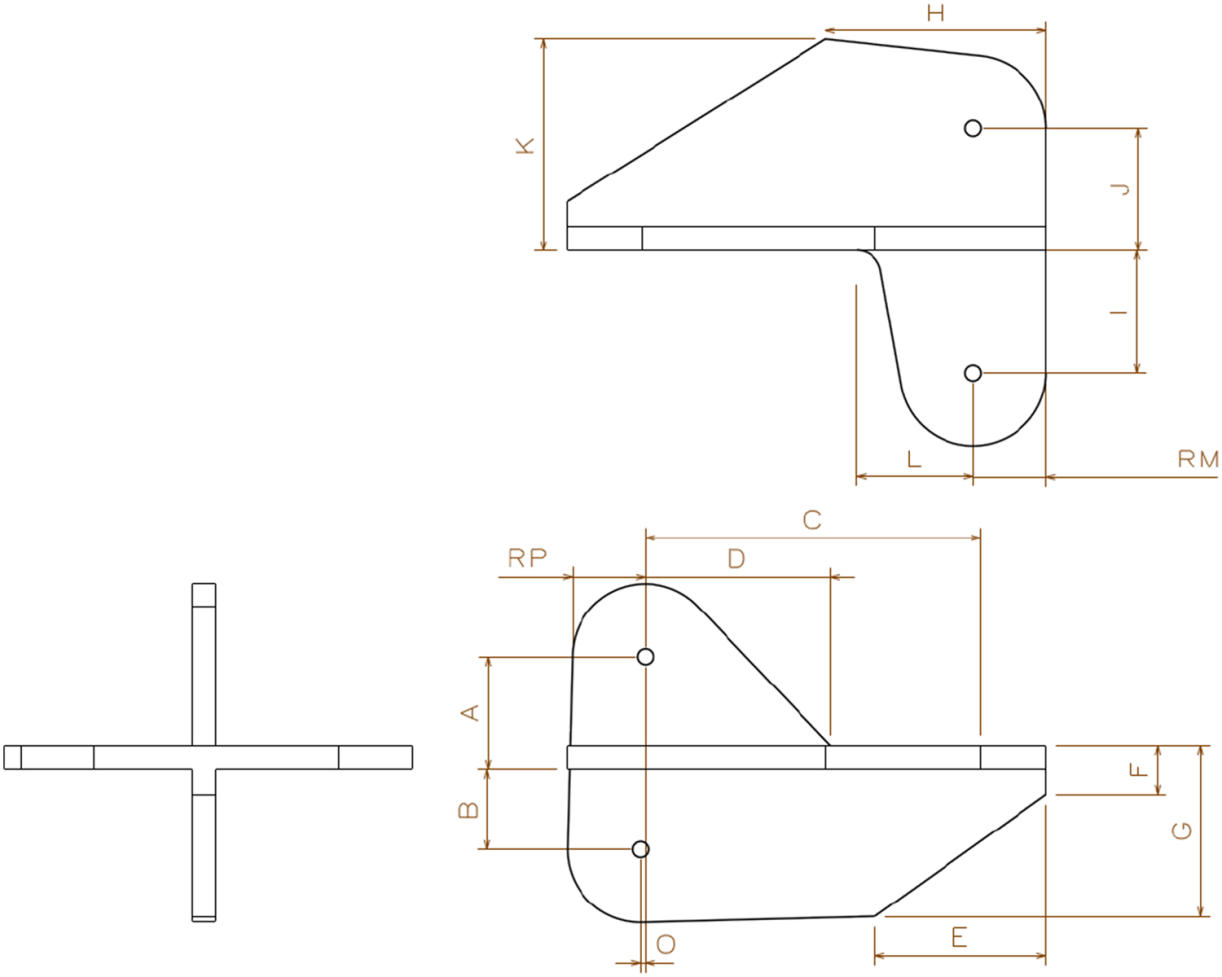

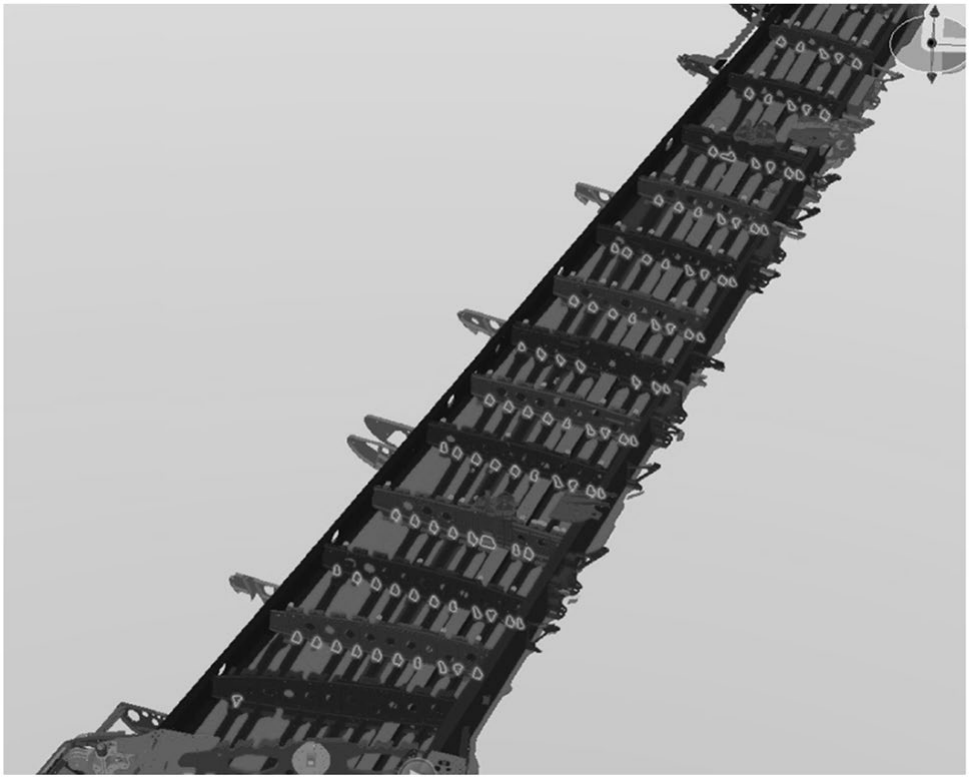

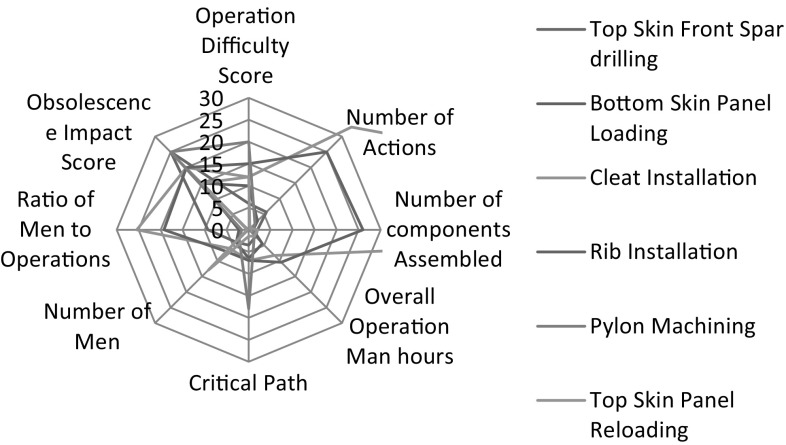

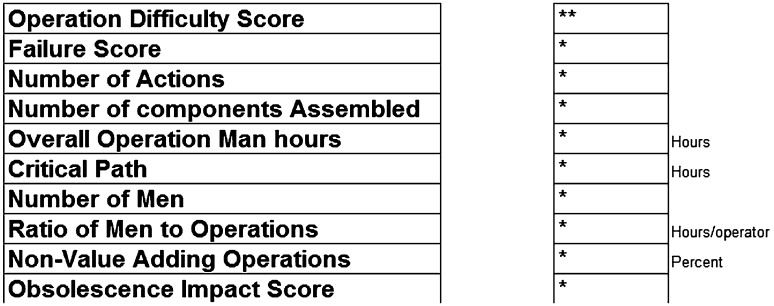
Showing the various design for assembly factors proposed in the methodology (no values shown)

One problem is the identification of which operations to analyse. The detailed analysis carried out for the cleat drilling operations will take a very long time to be carried out on every operation in wing box assembly. A shorter approach is, therefore, required to identify which operations should be analysed to this level of data. As discussed previously, the biggest driver in manufacturing and assembly improvement has always been the number of hours saved. A more recent driver that provides more direction is a reduction in the critical path. As such, the author has proposed the use of a two tiered approach. The first filter will organise operations based on the number of hours that lie on the critical path of wing box assembly. The second filter will organise the operations based on the total number of hours involved.

## Results and discussion

In order for the tool to provide meaning, it must be used in context. This requires data population of a number of other processes to compare against. The tool was used on six different processes. These processes were identified for analysis through a combination of recommendations from industrial and programme teams and some analysis of the assembly critical path to identify time consuming areas. The areas processed are as follows:

- top skin front spar drilling;
- bottom skin panel loading;
- cleat installation;
- pylon machining;
- rib installation;
- top skin panel reloading.

### Analysis

**Fig. 6:**
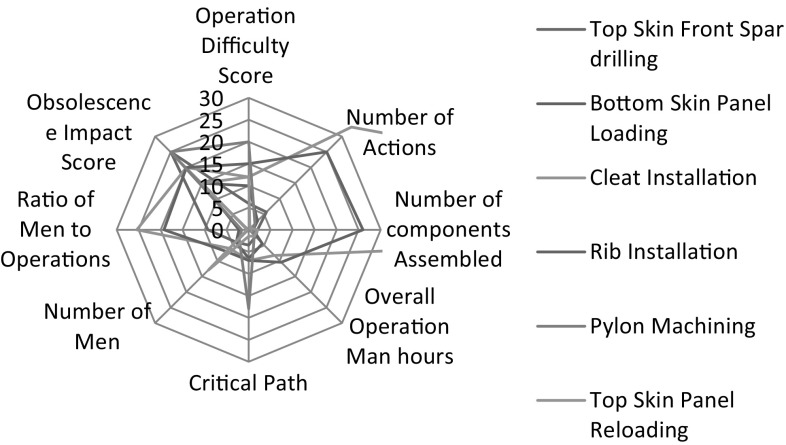
Showing the analysed processes compared against each other

Figure 6 shows a radar chart comparing the processes against each other. For the purposes of the chart, a normalisation process was carried out to plot the different factors on the same axis. Cleat installation is displayed in the analysis as a measure of the control. It is shown to score highly in a number of areas compared to the other processes displayed. This is a positive indicator as to the reliability of the tool as cleat installation is a process that has already been chosen for redesign by the business. The indicators explained in Sect. 11 can be split into two categories. The first are typical DFMA indicators and they are as follows:

- number of components;
- overall operation man hours;
- critical path time;
- ratio of operators to actions.

The second category are those indicators that relate directly to redesign for assembly of a product already in steady-state production. These are as follows:

- failure score;
- Operation Difficulty score;
- Obsolescence Impact score.

Each process displayed in the radar chart shown in Fig. 6 is thus analysed based on these categories.

### Top skin front spar drilling

This is the drilling process through the top skin and into the front spar. It involves the use of drilling templates and the progressive installation of temporary fasteners. The traditional DFMA indicators appear to illustrate that there is no need for redesign. However, the process is shown to have a high operation difficult score and a high OBI score. The operation difficulty score is high due to the requirement for specific tooling for back drilling to produce datum holes. This stems from the size of the panel components. As to the high OBI score, this is allocated for two separate reasons. The first is that geometric dimensioning and tolerancing methods (GD&T) have evolved in the past few decades and have even been implemented on newer programs. They have not been implemented on this legacy product and thus this datuming issue is present. In addition, the datum holes are required to be drilled in large flat areas that have no nearby features that can be datumed from. Thus, operators are required to datum from the other side of the panel, where there are ribs and other features to datum from.

The other contributing factor to the OBI score is that the technology used to carry out this process has been superseded. This process is now fully automated on newer aircraft.

### Bottom skin panel loading

This is the initial loading and wrapping of the bottom skin panels on to the ribs. The actual loading of the wing skins was found to be a relatively short process despite requiring highly skilled operators. The time consuming elements were in preparing the skins for loading.

This process was highlighted by stakeholders within the production organisation as an area for improvement. However, the OBI score it received is relatively low. This researcher investigated the disparity between the perceived problems of this process and its reality. The perception by the production teams was that there was a high failure rate due to the forces involved in wrapping the panels around the rest of the assembly and a perception that this induced necessary repositioning of the panel further along the assembly. This researched carried out a detailed study in this area in an attempt to identify DFA improvements. It was found that the assembly process itself was not problematic and that the process was not found to be obsolete. However, the compliant nature of the component, meant that movement during assembly was inevitable. An investigation into better forming processes was also launched. However, even with a more robust forming process, the size and nature of the component meant that this would have limited effect at this assembly stage.

This is a positive result for the tool as the tool indicated that limited improvement could be offered in this area.

### Cleat installation

This process has been discussed previously in this report. The process is already currently being addressed as it has been identified as a problem area. It is shown here to act as a measure of the success of the tool.

### Pylon machining

The pylon machining being addressed here is the drilling of a support plate in the pylon area. This process is shown to have a high critical path time. This is due to a number of factors related to the components themselves. The material in the pylon area is titanium, and as such, it is difficult to machine. In addition, locating parts around it and using fasteners to secure its location are more challenging due to the material being less compliant then aluminium. At the time that this was designed and launched into production, the processes that were implemented were the most efficient. However, as shown by a high OBI score, there are technologies available now that can offer a more efficient method of assembly. In addition, advances in GD&T ability have revealed that the tolerance requirement for this area does not need to be as tight as is specified. At pylon machining phase, the tolerance allowance allocated to the components is exceeded by the machining technology. This is due to an increase in the capability of the machining technology over the last few decades. Thus, if the tolerance at machining phase is made tighter, the tolerance at assembly phase can be relaxed achieving the same total tolerance allowance.

The operation difficulty score is also high. This stems from the fact that the uncompliant material is difficult to locate into position and this leads to operators having to adjust the assembly when parts move out of position.

A traditional DFA analysis will have indicated that this area was indeed difficult but that it was necessary and there was no need to change anything. The redesign indicators inform the engineer that there is potential for improvement.

### Rib installation

This is the process of assembling the ribs to the front and rear spars. Traditional DFA indicators point to a reassessment of the area as it is a process that lies directly on the critical path and also has a high number of components. However, a traditional DFA assessment would also indicate that the parts are necessary and that they are required to be separate components. The redesign indicators tell a different story. A high operation difficulty score and a high OBI score point towards an opportunity in this process. The high OBI score is due to the same process being upgraded on a newer aircraft program through a modern GD&T analysis. The new process involves a determinant assembly approach and is a more cost effective way of installing the ribs. The introduction of such a process would also reduce the operation difficulty score. This is because when the main reasons that this was high were because of the difficulty in locating the ribs and adjusting their position.

### Top skin panel reloading

This operation involves relocating the top skin. The top skin is removed from the assembly after it has been drilled in order for cleaning and deburring to take place. It is then relocated onto the structure using the same original method of location. This operation is similar to the process of bottom skin panel loading discussed previously and shares many of the same challenges. The reason it was also included in this assessment was due to a recommendation by the production teams. The reasoning was that this was a more challenging process then the initial loading onto the wing box assembly as the holes that have been drilled are now required to line up. This is represented by a slightly higher operation difficulty score as readjustment of the assembly was occasionally required. However, as with the bottom skin loading, the operators carrying out this process are highly skilled and are usually able to locate the skin panel efficiently. An over reliance on those operators may perhaps create a bottleneck if they are needed elsewhere but this offset by the cost of developing a technological solution.

## Summary and recommendations

Acknowledgement has been added that the general methodology is a general methodology and can be applied in any redesign scenario; however, the tool for every manufacturing system requires unique development.

The tool demonstrated positive initial success. The tool was able to highlight that the cleat installation process was indeed problematic and that it suffered from obsolescence. This corresponded to the business decision to carry out a redesign in that area. In addition, the tool pointed towards two more areas that could benefit from redesign for assembly projects. These two projects were also highlighted by the factory as potentially profitable. The other areas that were analysed were not deemed profitable, and this was the same view of the factory. The one area of discrepancy was the loading of the wing skins. The plant decided to further investigate the form of the wing skins throughout production. However, this proved to be evidence in favour of the methodology and the tool as after the investigation was carried out, no obvious benefit could be identified.

The redesign indicators were able to highlight processes for project consideration, where traditional DFA analysis could not. Obsolescence analysis and operation difficulty assessment were shown to be useful tools for analysis in established production systems.

A deficiency in the tool is that the tool only studies processes in isolation. A process is very likely to be affected by previous processes on the same assemblies or components. This was demonstrated through the investigation into the assembly of panels where a better understanding of the area could only be achieved through a root cause analysis of the component from its machining phase to its assembly phase. Future development of the tool will attempt to factor in component and time-related elements.

It is important to acknowledge that the tool is specific to this scenario. The methodology, demonstrated in Fig. 1, is a general methodology that can be applied for any redesign scenario. However, every manufacturing system will require the development of its own tailored tool. Even if this methodology was to be used for another stage of production of the same wing box, the tool would require adaptation to that requirement.

It is typically assumed that critical path is the most prominent factor in making decisions with regard to manufacturing and this is why it is included as a part of this analysis. However, in a redesign setting, there are a number of factors that might be prioritised at a particular time. For example, if delivery dates have been established, then reducing total operation hours is perhaps more profitable for that product than a reduction in critical path. This tool enables the engineer to decide on which project to analyse based on the current factors that a product is being subjected to.
